# ID 157 - Estratégias de um Nats para Disseminação da Avaliação de Tecnologias no Contexto do SUS

**DOI:** 10.5327/2237-9622.2025.v34s1.157

**Published:** 2025-11-25

**Authors:** Daniela Oliveira de Melo, Andréa da Silva Dourado, Nanda Isele Gallas Duarte, Morgana do Canto Pesenti

## Abstract

**Introdução:**

Instituída em 2011 com o objetivo de promover a difusão da Avaliação de Tecnologias em Saúde (ATS) no âmbito do SUS, a Rede Brasileira de Avaliação de Tecnologias em Saúde (Rebrats) é formada por Núcleos de Avaliação de Tecnologias em Saúde (Nats) para incentivo, desenvolvimento e produção de pesquisas e estudos no campo da ATS. Membro da Rebrats e comprometido com a articulação entre ensino, pesquisa e gestão em uma universidade pública federal, o Nats da Unifesp Diadema (NUD) possui como missão o desenvolvimento e o fomento da disseminação de conhecimento científico nas áreas de ATS e políticas públicas para subsidiar a tomada de decisão e a formação de recursos humanos em saúde. Assumindo suas premissas de rigor metodológico, transparência, responsabilidade social e compromisso com o Sistema Único de Saúde (SUS). O NUD identificou a necessidade na formulação de estratégias que contribuam para fortalecimento da rede nacional de ATS por meio do aumento da visibilidade da produção dos Nats e do efeito do trabalho desses grupos no sistema de saúde. Nesse sentido, optou pela formação e ampliação de áreas complementares, promovendo a integração entre a ATS e a comunicação social. Considerando um público potencial em ATS que varia de profissionais especialistas a universitários, e a utilização atual massiva de redes e mídias sociais, foram adotadas estratégias para promoção da transmissão de informação e da tradução do conhecimento nas redes sociais do núcleo por meio de categorias temáticas, visando divulgar eventos sobre ATS (NUD Participa), disseminar notícias relevantes para área (NUD Repercute), aumentar o alcance de publicações em ATS (NUD Publica) e disponibilizar conteúdos pedagógicos sobre ATS (Hora do Estudo). Foi, ainda, desenvolvido e publicado um site (nud.unifesp.br) como canal facilitador para veiculação da organização interna do núcleo, bem como de suas parcerias, divulgação de pesquisas e publicações técnico-científicas (como o Saber-SUS, realizado no âmbito do Programa de Pesquisa para o SUS, e os produtos de ATS subsidiados pelo Dgits) e introdução a termos relevantes em ATS, por meio de um glossário. Durante o V Congresso da Rebrats espera-se apresentar as iniciativas de comunicação adotadas pelo NUD por meio da exposição das redes sociais e do site do núcleo, bem como estimular a discussão sobre estratégias que podem ser incentivadas para impulsionar a disseminação da ATS e destacar o trabalho dos Nats em parceria com a Rebrats.

**Método:**

A dinâmica será dividida em diferentes momentos expositivos, sendo necessários equipamentos para projeção de imagem de tela de desktop, idealmente com acesso à internet para exibição redes sociais e sites. A apresentação deve considerar as seguintes etapas: (1) contextualização e problemática; (2) demonstração das iniciativas de divulgação em redes sociais, por meio da apresentação de categorias temáticas, enfatizando seus objetivos e público-alvo; (3) apresentação dos recursos e informações disponibilizados no site para promoção da disseminação da ATS.

